# Optimizing artificial meniscus by mechanical stimulation of the chondrocyte-laden acellular meniscus using ad hoc bioreactor

**DOI:** 10.1186/s13287-022-03058-w

**Published:** 2022-07-30

**Authors:** Mehri Shadi, Tahereh Talaei-Khozani, Mahsa Sani, Radmarz Hosseinie, Hossein Parsaei, Zahra Vojdani

**Affiliations:** 1 Tissue Engineering Lab, Department of Anatomical Sciences, School of Medicine, Shiraz University of Medical Sciences, Shiraz, Iran; 2 Histomorphometry and stereology research Center, Shiraz Medical School, Shiraz University of Medical Sciences, Shiraz, Iran; 3 Tissue Engineering Lab, Department of Anatomcal sciences, School of Medicine, Shiraz University of Medical Sciences, Shiraz, Iran; 4 Tissue Engineering Department, School of Advanced Medical Science and Technology, Shiraz University of Medical Science, Shiraz, Iran; 5 Department of Mechanical Engineering, College of Engineering, Fasa University, Fasa, Iran; 6 Department of Medical Physics and Engineering, School of Medicine, Shiraz University of Medical Sciences, Shiraz, Iran; 7 Laboratory for Stem Cell Research, Department of Anatomical Sciences, School of Medicine, Shiraz University of Medical Sciences, Shiraz, Iran

**Keywords:** Decellularized extracellular matrix, Meniscus, Mechanical stress, Bioreactor

## Abstract

**Background:**

Tissue engineering focuses on reconstructing the damaged meniscus by mimicking the native meniscus. The application of mechanical loading on chondrocyte-laden decellularized whole meniscus is providing the natural microenvironment. The goal of this study was to evaluate the effects of dynamic compression and shear load on chondrocyte-laden decellularized meniscus.

**Material and methods:**

The fresh samples of rabbit menisci were decellularized, and the DNA removal was confirmed by histological assessments and DNA quantification. The biocompatibility, degradation and hydration rate of decellularized menisci were evaluated. The decellularized meniscus was injected at a density of 1 × 10^5^ chondrocyte per scaffold and was subjected to 3 cycles of dynamic compression and shear stimuli (1 h of 5% strain, ± 25°shear at 1 Hz followed by 1 h rest) every other day for 2 weeks using an ad hoc bioreactor. Cytotoxicity, GAG content, ultrastructure, gene expression and mechanical properties were examined in dynamic and static condition and compared to decellularized and intact menisci.

**Results:**

Mechanical stimulation supported cell viability and increased glycosaminoglycan (GAG) accumulation. The expression of *collagen-I* (COL-I, 10.7-folds), *COL-II* (6.4-folds), *aggrecan* (AGG, 3.2-folds), and *matrix metalloproteinase (MMP3*, 2.3-folds) was upregulated compared to the static conditions. Furthermore, more aligned fibers and enhanced tensile strength were observed in the meniscus treated in dynamic condition with no sign of mineralization.

**Conclusion:**

Compress and shear stimulation mimics the loads on the joint during walking and be able to improve cell function and ultrastructure of engineered tissue to recreate a functional artificial meniscus.

## Introduction

The meniscal disorders occur due to abnormal development and traumatic and sports injuries that could be associated with ligament tears around the knee joint and tibial plateau fractures. Patients with meniscus injuries suffer from pain, swelling, and movement disorders, which often need surgical therapy. If no treatment is achieved, patients will be affected by the risk of degenerative changes of the knee joints [1, 2].

The menisci are two semicircular, wedge-shaped fibrocartilages located in the knee joint between the femoral condyle and tibial plateau. The main functions of the meniscus are to maintain stability, redistribute and transfer load through the joint, and absorb shock. Since the main part of the meniscus is avascular, any meniscus injury cannot be completely recovered, and as a result, it may be associated with debility and pain [3, 4].

Surgical techniques and allograft transplantation are the first approaches for meniscal treatment, but these therapeutic operations have inherent limitations in clinical practice including the shortage of donated organs and immune system rejection. This highlights the need for tissue engineering to provide an alternative usable solution [5, 6]. Tissue engineering is a multidisciplinary field to regenerate new tissues using a combination of cells, scaffolds, growth factors, and biochemical and mechanical stimuli. Bioengineering technologies mimic the natural structure of the tissues to fabricate artificial organs since it emphasizes mimicking not only the tissue biochemistry, but also mechanical and geometric properties [4].

Meniscus contains COL-I and II (mostly types I), proteoglycans, glycoproteins and cells [7]. Collagen fibers are organized in the circumferential and radial direction to bear tensile and compression loads and absorbs shock in response to joint movement. The meniscus has displayed biphasic and viscoelastic behaviors when subjected to stress or strain [8]. In scaffold-based approaches, the biomaterials and growth factors have complementary effects on the cell behaviors. Meniscal engineered scaffolds have been fabricated by either synthetic or natural materials. The entire decellularized meniscus has the advantage of organized compatible architecture that provides a natural microenvironment for meniscal cells. This type of scaffold displays good biocompatibility, biomechanical and regenerative properties and presents a promising approach to the functional reconstruction of the injured menisci [9]. Decellularized meniscus in ovine [10], porcine [11], and rabbit [12] showed proper porosity, fiber organization, and zonation. Besides, it induced chondrogenic differentiation in the mesenchymal stem cells (MSCs) [11].

Normal development and function of the meniscus depend on mechanical stimulation. In vivo mechanical stimulation has been shown to induce meniscus growth and ECM remodeling to accommodate the force [13]. Various types of bioreactors have been developed to simulate the physiologic stresses and may provide appropriate in vitro conditions for meniscal regeneration. Different categories of cartilage bioreactors have been designed to recapitulate the artificial meniscus [14]. Through exerting combined compression and tension forces, bioreactors would be able to improve the biochemical and biomechanical properties of the tissue-engineered meniscus and align their collagen fibers [15].

Mechanical stimulation has been applied to the encapsulated chondrocytes in different biomaterials to fabricate artificial meniscus. Dynamic intermittent compression has been applied on fibrochondrocytes loaded into alginate, and it was found that prolonged compression could initiate a catabolic cellular response and decrease ECM retention compared to a short time loading [16]. In another study, static and dynamic compression of the chondrocytes in polyglycolic acid stimulated chondrogenesis. Although mechanical loading had a decreasing effect on glycosaminoglycan (GAG) concentration and ECM synthesis rates, it elevated the DNA and collagen fibers content [17]. A significant decrease in COL-II and increase in GAG content have been reported by applying flow shearing force on the chondrocytes encapsulated in polyglycolic acid [18].

In the other study, chondrocytes loaded in a hydrogel formed by collagen type I expressed a lower amount of aggrecan without any significant changes in the collagen type II/collagen type I mRNA ratio. The mechanical stimulation has also detrimental impacts on mechanical properties of the hydrogel. It has been suggested that the hyrogles have no capability to enhance the mechanical properties by exposure to mechanical stimulation [19]. This controversy revealed that both the type of biomaterial and its architecture and mechanical stimulation are critical for designing a functional artificial meniscus [18].

Most of studies applied mechanical forces on the scaffolds formed from synthetic or natural biomaterials and these scaffolds can’t provide an exact biomimicry 3D structure of native meniscus. Besides, most of the used hydrogels in those study are too weak to bear mechanical load. Decellularized meniscus provides an appropriate biomimicry scaffold with good mechanical strength that bear both of compress and shearing forces. These mechanical stimulations help recapitulating functional cell loaded meniscus. In the present study, we used decellularized meniscus as a natural ECM for the chondrocytes and exposed them to compress and shearing forces to mimic the tissue mechanochemical microenvironment and fabricate a bioartificial meniscus.

## Materials and methods

### Tissue collection and decellularization

**Fig. 1:**
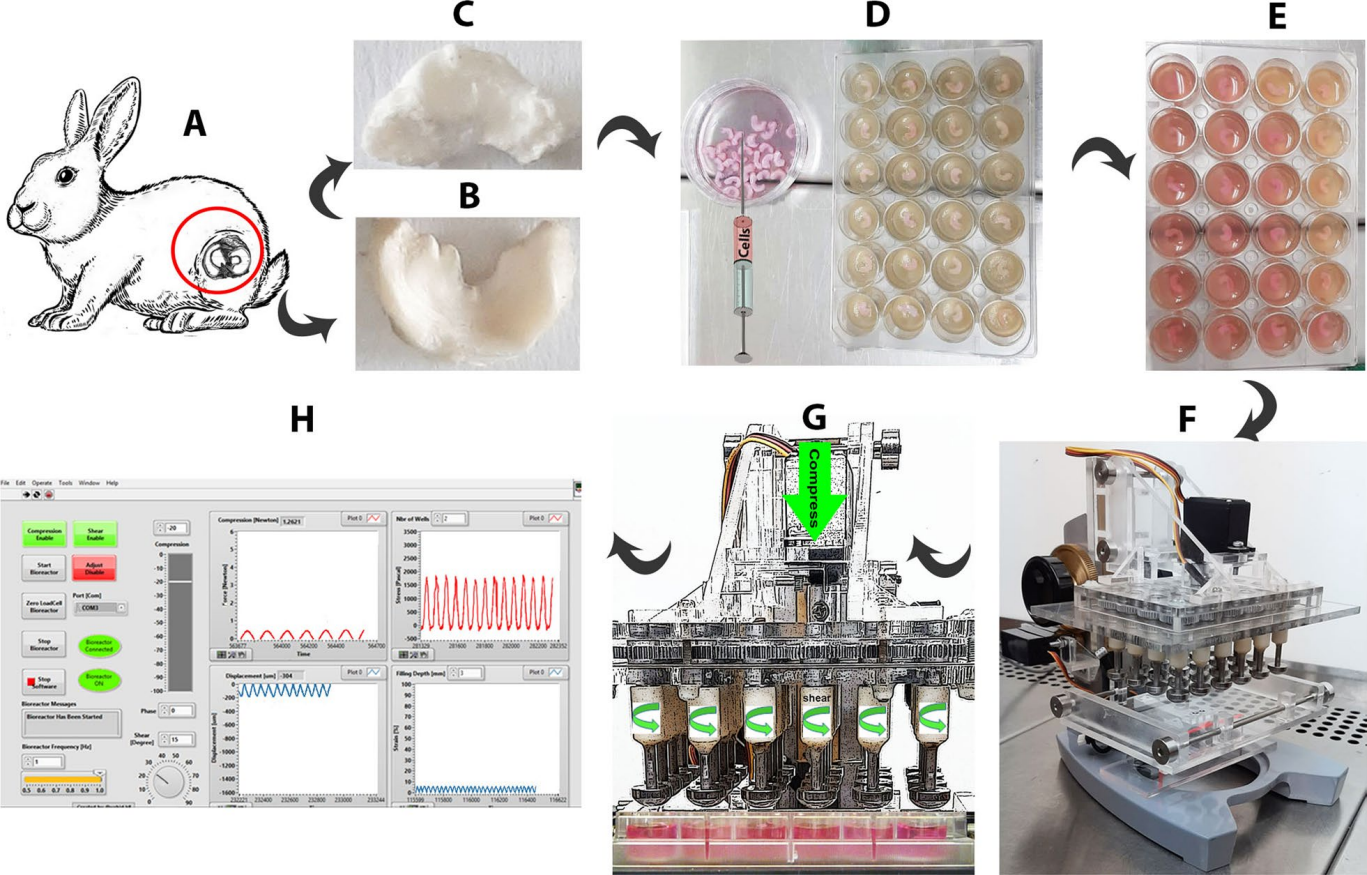
Whole menisci were harvested from adult rabbit knee joints (**A**) native (**B**), and decellularized meniscus (**C**). Cell-laden scaffolds immobilized at the center of the wells of a 24 well-plate containing agarose gel (**D**), supplemented with culture medium (**E**). The scaffolds were exposed to the biaxial bioreactor (**F**) that applied shear and compression loading (**G**). Ad hoc designed software for controlling the bioreactor (**H**)

The fresh samples of the menisci were harvested from male New Zealand white rabbits (Fig. 1A). The animal treatment was in accordance with the Shiraz University of Medical Sciences guidelines, and the experimental design was approved by the ethics committee (IR.SUMS.REC.1400.085). The samples were washed with phosphate buffer saline (PBS) and stored in − 20 until further usage.

Whole menisci were immersed in 10 mM Tris–HCl buffer for 24 h followed by four cycles of freezing and thawing for 48 h. Then, the thawed tissues were incubated in 0.25% trypsin (Sigma-Aldrich) and immersed in 1%Triton X-100 (Sigma, USA), each step for 24 h. The menisci were then placed in 1.5% Sodium lauryl ether sulfate (SLES, Kimia Sanaat Ataman Co., Iran) followed by 0.3% ethylenediaminetetraacetic acid (EDTA, Parstous, Iran), each step for 48 h. Thereafter, they were stirred in 1% Triton X-100 for 24 h; finally, the samples were treated with 0.05% trypsin–EDTA for 24 h. To eliminate cellular debris and increase porosity, we incubated the samples in a solution containing 2% Triton X-100 and 1.5% peracetic acid for 48 h.

### Characterization of decellularized meniscus

Intact and decellularized menisci were fixed in 4% paraformaldehyde (Sigma-Aldrich, USA) and embedded in paraffin. Serial sections of 5-μm thickness were stained with hematoxylin and eosin and Hoechst for confirmation of efficient cell removal.

To confirm DNA removal, we freeze-dried the samples of both intact and decellularized scaffolds and weighed them (25 mg) for DNA quantification assay. DNA was then isolated with a commercially available Tissue Genomic DNA Extraction mini kit (FavorPrep, Taiwan) according to the manufacturer’s instructions, and the DNA yield (ng/μL) was quantified by spectrophotometer (Nanodrop Technologies Inc., Wilmington, DE, USA) at an optical density (OD) of 260 nm.

Scanning electron microscopy (SEM) was performed to evaluate the ultra-architecture of the scaffold. To do this, they were fixed with 2.5% glutaraldehyde (Sigma-Aldrich, St. Louis, MO, USA), rinsed in 0.1 M PBS (pH 7.4), and then lyophilized by a freeze-drier (Christ Alpha 2–4 LD-plus, Osterode am Harz, Germany). The samples were coated with a thin layer of gold, using Q150R–ES sputter coater (Quorum Technologies, London, UK) and imaged by a VEGA3 microscope (TESCAN, Brno, Czech Republic).

For ECM preservation evaluation, Masson Trichrome, periodic acid schiff (PAS), Alcian blue (pH 2.5), and Gomori's aldehyde-fuchsin staining were used to visualize the collagen fibers, neutral carbohydrates, GAGs, and elastic fibers, respectively.

### Degradation rate

Three lyophilized decellularized scaffolds were placed in 1% trypsin in PBS (pH7.2) at 37 °C for up to 4 weeks. The samples were dried and weighed every 24 h for one week and every week for one month. The weight loss was calculated by the following formula:

$${\text{Weight loss }}\left( \% \right)\, = \,\left( {W_{o} \, - \,W_{t} } \right)/W_{o} \, \times \,100$$

where *W*_o_ is the initial dried weight and W_t_ is the dried weight of the degraded sample.

### Swelling ratio evaluation

The lyophilized meniscal scaffolds were weighed and immersed in distilled water at room temperature. Weight was continuously measured and recorded until it reached a plateau. The swelling ratio was calculated using the following equation,  where W is the weightof freeze-dried construct and W_0_ is weight of the wet construct: 

$$\left( {wt.\% } \right)\, = \,\left( {W - W_{o} } \right)/W_{o} \, \times 100$$

### Biocompatibility evaluation

The biocompatibility of the decellularized menisci was evaluated by implanting them into the subcutaneous tissues of six Sprague–Dawley rats. Three rats were randomly sacrificed after euthanasia at 1 week and 1 month post-implantation. Menisci and the surrounding tissues were removed and subsequently fixed in 4% paraformaldehyde for 48 h. The paraffin-embedded sections at 5 μm thickness were stained with hematoxylin and eosin (H&E).

### Experimental design

The total decellularized menisci were lyophilized and re-sterilized by UV. Chondrocyte cell line C28/12 (Pasteur Institute) was injected with a 31-gauge insulin syringe at a density of 1 × 10^5^ per scaffold (average size, 1.5 × 3 × 8 mm) in Dulbecco's Modified Eagle Medium (DMEM) supplemented with 10% Fetal Bovine serum (FBS), 1% Penicillin /Streptomycin, 1% L-glutamate and cultured at 37 °C and, 5% CO_2_ for 1 week to allow the cells to penetrate into the decellularized scaffold. Twenty-four cell-seeded scaffolds were kept under static conditions without compression and shear stimulation. The other set of cell-laden scaffolds were immobilized at the center of the wells containing 7% agarose gel. After one day, the constructs were subjected to 3 cycles per day of dynamic compression and tangential shear loads (1 h of 5% strain with a displacement of 200 μm amplitude, ± 25°shear at 1 Hz followed by 1 h rest) for 2 weeks (3 days/week, Fig. 1BG). The medium was changed every 2 days [20, 21].

### Bioreactor

An ad hoc bioreactor was designed to apply a biaxial mechanical load (compress and shear) to the cell-laden engineered meniscal scaffolds. The bioreactor is small enough (22 cm high × 18 cm × 20 cm) to be placed within a standard laboratory incubator for a long-term culture with a temperature-controlled environment and standard buffering systems (pH7.2). The device contains two main modular subsystems: the compression and rotational shear mechanisms. Each mechanism has a separate servomotor that was used to produce motion independently. The gearbox of the shear mechanism contains 24 all-meshed aluminum gears, each one being attached and aligned with a stainless steel ‘condyle’, which is precisely tuned for 24-well plate, and performs vertical linear movement (compression), and rotational motion around its axis, (shear).

The compression and shear subsystems of the device produce harmonic, i.e., sinusoidal motions separately. The compression can achieve maximum amplitude of 1 mm and frequency up to 1 Hz. The precision of the compression motion is about 0.01 mm. The shear motion has maximum amplitude of 180 degrees with a maximum frequency of 1 Hz. The precision of shear motion is about 1.5 degrees. The capacity of the load cell is 6 N with the precision of 0.1 N. The computer software for the operation and control of the apparatus was developed using the LabView package (Fig. 1H).

### Cytotoxicity assay

The cell viability was evaluated by culturing the chondrocytes at the same condition as described above for 4, 8, and 12 days. The cells in the scaffolds that were exposed to dynamic and static conditions along with 2D conventional cultures as the control were treated to 1 mg/mL 3-(4, 5-dimethyl thiazolyl-2)-2, 5-diphenyltetrazolium bromide (MTT, M5655; Sigma–Aldrich) for 3 h at 37 °C and 5% CO_2_. Formazan was eluted by adding dimethyl sulfoxide (Sigma–Aldrich) for 20 min. The optical density of the eluted MTT was measured by a spectrophotometer at 590 nm.

### GAG quantification assay

GAG contents of the lyophilized decellularized meniscus, the recellularized scaffolds under dynamic and static conditions, and intact tissue were analyzed using the dimethyl methylene blue (DMMB) assay. The dye solution contained 16 mg DMMB, 3.04 g glycine, 1.6 g NaCl, and 95 mL of 0.1 M acetic acid reconstituted per one liter of distilled water, and the pH was adjusted to ~ 3. 100 mg of each tissue were minced and digested with 0.5 mg/mL proteinase K at 56 °C overnight to quantify the GAG. Twenty µL of the digested samples were added to 200µL of DMMB in a 48-well plate. After pipetting, absorbance was immediately measured at 656 nm, using a microplate reader [22].

### Scanning electron microscopy (SEM)

Samples of the intact, decellularized, and recellularized menisci with and without mechanical stimulation were prepared for SEM, as described above. The pore size of the scaffolds was evaluated by ImageJ software (http://mac.softpedia.com/get/Graphics/ImageJ.shtml).

### Alkaline phosphatase assay

Alkaline phosphatase (ALP) activity was evaluated by a commercial kit (Pars Azmoon, Iran) according to the manufacturer’s instruction in both 3D static and dynamic conditions and compared to enzyme activity in 2D conventional monolayer cell culture.

### Immunohistochemistry

Frozen sections at a thickness of 7 μm were prepared from the cell-laden scaffolds in both dynamic and static conditions. Blocking of endogenous peroxidase activity and non-specific binding sites was done by incubating the slides in 0.3% H_2_O_2_ in methanol, and PBS containing 10% goat serum and 5% BSA, respectively. The sections were then incubated in biotinylated anti-collagen type I antibody (1:250; ab6577) overnight at 4 °C. After washing with PBS, streptavidin-HRP (1:10,000; Abcam, USA; ab7403) was added to each section and incubated for 20–30 min at room temperature followed by incubating in diaminobenzidine (DAB, Dako) for 15 min.

The matched series of slides were also incubated in Collagen type II primary antibody (1:100, (ab634712) followed by incubating in the linker (Diagnostic BioSystems-PVP1000D) for 15 min and then in polymer (Diagnostic BioSystems-PVP1000D) for 30 min. Color development was done by adding DAB. Both immunostainings were counterstained with hematoxylin.

### Quantitative RT-PCR

Five duplicated samples for each group at each point of time were subjected to real-time quantitative RT-PCR. Scaffolds were digested with 0.5 mg/mL of proteinase K at 56 °C overnight. The total RNA of the samples was extracted by standard RNX plus (Sinaclon, Sinohe Biotech, Iran), and the concentration and purity of the RNA were checked with a NanoDrop Spectrophotometer. The absorbance ratio (260/280 nm) > 1.8 was used to ensure the purity of the extracted RNA. The mRNA was reverse transcribed into cDNA by a cDNA Synthesis Kit (Fermentas; Thermo Fisher Scientific, Inc). Reactions were conducted at 95 °C for 15 min, followed by 40 cycles of 95 °C for the 30 s, 58 °C for 30 s, and 72 °C for 30 s. Gene expressions of *COL-I*, *COL-II*, *AGR*, and *MMP-3* were quantified by real-time PCR on a Rotor-Gene Q thermocyclers (RGQ, QIAGEN GmbH, Hilden, Germany), using SYBR Green Low ROX Master Mix (Amplicon, Brighton, UK). The sequence of specific primers of target genes is shown in Table 1. The mRNA levels of *COL-I*, *COL-II*, *AGG*, and *MMP3* were all normalized to the value of Beta-actin at the corresponding time points.

**Table 1:** Primer sequences used in the assessment of gene expression by real-time polymerase chain reaction analysis

Gene	Primer Sequence (5'-3')	Sizes (bp)
B-actin	Forward: GCCTTTGCCGATCCGC Reverse: GCCGTAGCCGTTGTCG	90
COL-I	Forward:CGGCTCCTGCTCCTCTTAG Reverse: GGGCTCGGGTTTCCACACG	150
COL-II	Forward:GATGGCTGCACGAAACATAC Reverse:CATGGGTGCAATGTCAATGAT	99
AGR	Forward: TTCTGCTTCCGAGGCATTT Reverse: CAGCAGTTGTCTCCTCTTCTAC	148
MMP3	Forward:TGGACAAAGGATACAACAGGGA Reverse: GTGAGTGAGTGATAGAGTGGGTA	125

### Mechanical tests

To analyze the mechanical properties of the scaffold, we performed tensile and compression tests. The decellularized, mechanically stimulated, and non-stimulated scaffolds and intact menisci (n = 3 for each group) were fixed in a testing machine (Santam, STM-50, Iran) to determine the tensile and compression modulus. Samples with a width of 3 mm, length of 8 mm, and thickness of 1.5 mm were measured by a Digital Vernier Caliper (Louisware, Dubai). Then, the samples were loaded to disrupt at the rate of 3.5 mm/min. The tensile and compression moduli were defined as the slope of the liner segment of the strain–stress curve [23].

### Raman confocal microscopy

The Raman spectra of the decellularized meniscus were evaluated. The laser power level was 50 mW using the excitation laser wavelength of 785 nm. In this study, the Raman spectra were analyzed in the range of 300 to 1800 cm^−1^ with a resolution of 4 cm.^−1^[24]

## Results

### Decellularization efficiency

**Fig. 2:**
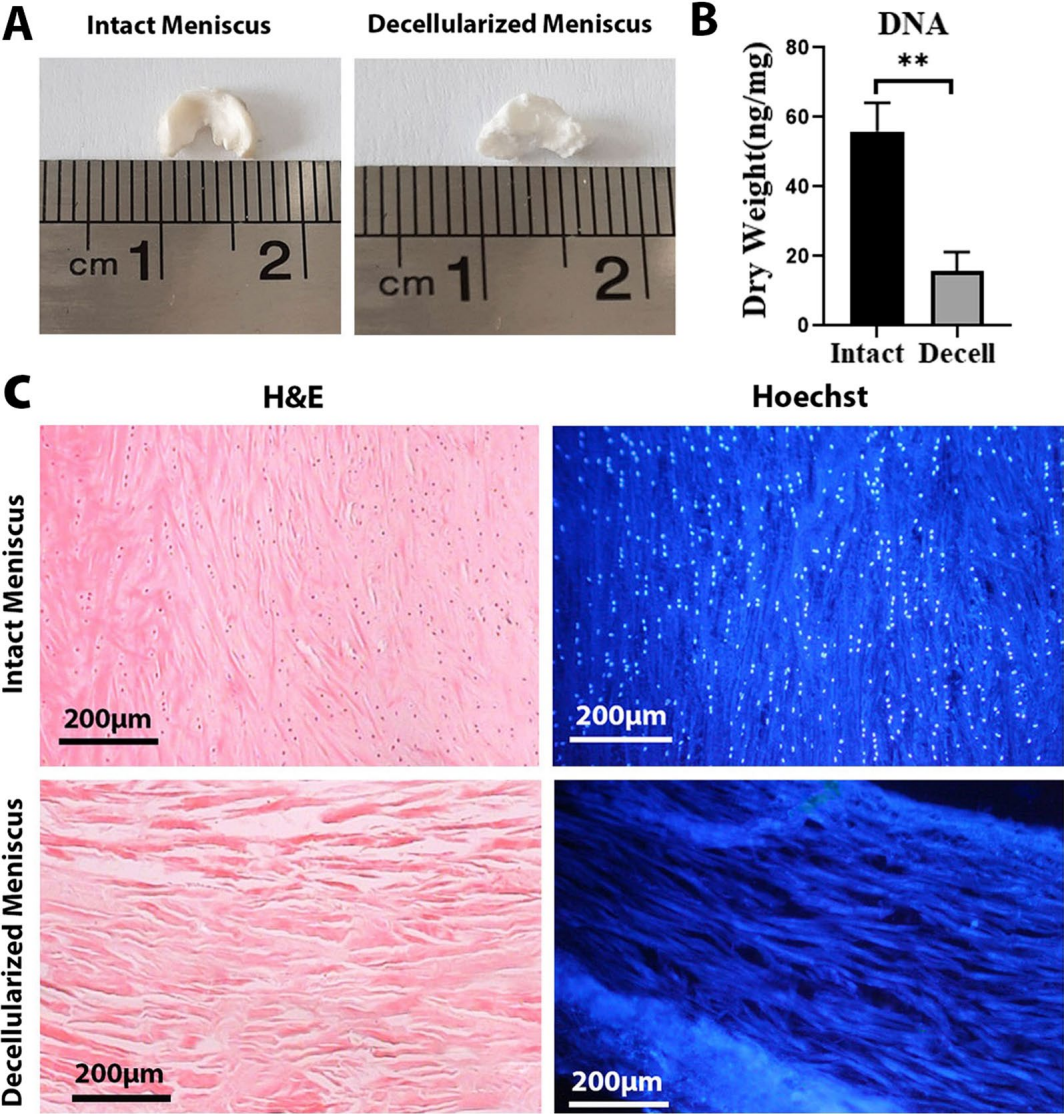
**A** Gross appearance of the decellularized and intact menisci. **B** Comparing the DNA content in decellularized and intact menisci (25 mg) *p < 0.01 (**C**) Histological staining (H&E and Hoechst) of the intact and decellularized menisci

Gross observation of the lyophilized decellularized scaffolds revealed that they preserved the shape (Fig. 2A). DNA quantification assay revealed that the DNA content in the decellularized meniscus was 15.73 ± 5.51 ng/mg per dry weight, which is significantly lower than that in the native meniscal tissue (55.87 ± 8.22 ng/mg dry weight, *P* = 0.002, Fig. 2B) Analysis of the cell removal for the decellularized meniscal scaffolds was completed using H&E and Hoechst staining. Histological analyses showed that the nuclei in the decellularized scaffolds were removed properly. However, a negligible number of nuclei were observed in some sections (Fig. 2C).

### SEM analysis

**Fig. 3:**
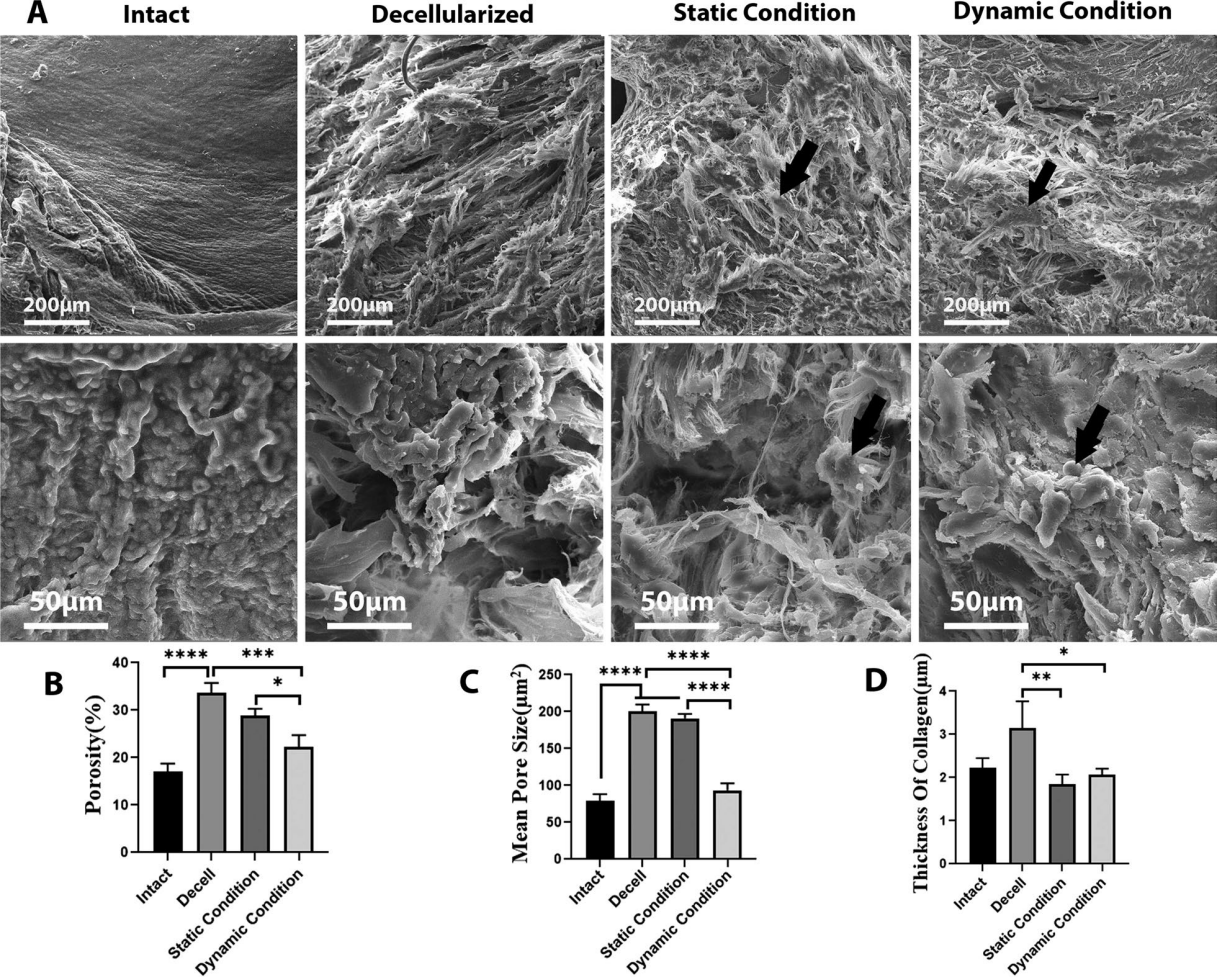
**A** SEM images of intact, decellularized meniscus, and the recellularized scaffolds in static and dynamic conditions. Higher magnification of each one was depicted in lower raw of SEM micrographs. Arrows show the chondrocytes seeded on decellularized menisci under static and dynamic conditions. The graphs compare the (**B**) porosity (%), (**C**) pore size(µm^2^) and (**D**) collagen fiber thickness of intact and the other treated groups

Porosity, pore size, and orientation of the collagen fibers in decellularized and intact menisci were evaluated by analyzing the SEM images (Fig. 3A) using ImageJ software. Findings showed a significant increase in the porosity in decellularized (33.63 ± 2.13%) compared to the intact meniscus (17.13 ± 1.64%, *P* = 0.00007). The force exposure of the recellularized scaffolds markedly decreased the porosity compared to both recellularized scaffolds in the static conditions (*P* = 0.04) and decellularized meniscus (*P* = 0.01, Fig. 3B). The mean value of the pore size was also higher in decellularized (200.3 ± 9.43µm^2^) than intact (78.93 ± 9.22µm2) tissues (*p* = 0.000006). The mean value of the pore size also significantly diminished in dynamic conditions compared to the static conditions and decellularized meniscus (*P* = 0.00003, *P* = 0.00001, respectively, Fig. 3C).

Table 2 summarizes the goodness and the angles of fit in the scaffolds expose to dynamic and static conditions as well as cell free decellularized and intact meniscus. The goodness of fit revealed more aligned fibers in the intact tissue compared to the decellularized one. Therefore, it seems that decellularization led to some extent of disorganization in the fibers. The collagen fibers under static and dynamic conditions were slightly more disordered than intact meniscus. Therefore, the fibers in dynamic condition were more aligned than static condition. Decellularized meniscus contained non-significantly thicker fibers than the intact tissue (*P* = 0.05). A significantly thicker fiber was formed in decellularized tissue compared to the scaffolds in both static (*P* = 0.008) and dynamic (*P* = 0.02) conditions (Fig. 3D)**.** Comparing the cell loaded menisci in static and dynamic conditions revealed that the force exposure had no significant impact on the fiber thickness.

**Table 2:** Fibers orientation parameters

Orientation parameters	Intact	Decellularized menisci	Static condition	Dynamic condition
Goodness Of Fit	0.97	0.89	0.83	0.87
Direction Of Fibers	14.49°	25.70°	59.97°	-13.98°

### In vivo biocompatibility evaluation

**Fig. 4:**
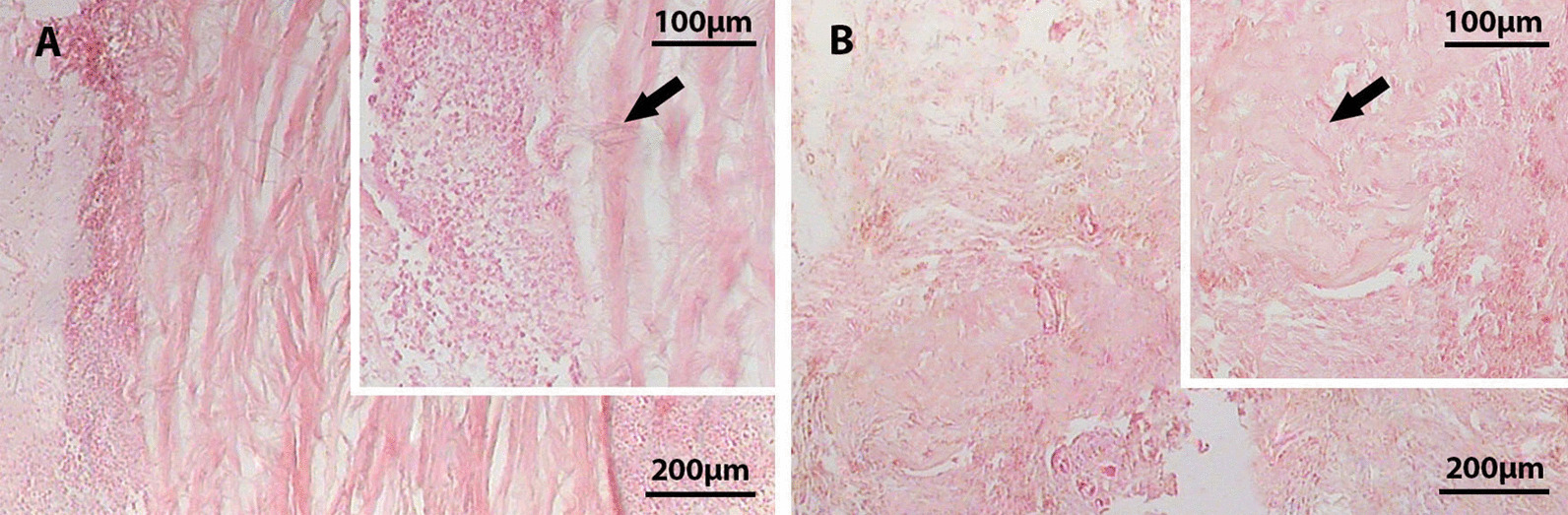
H&E staining of the in vivo implanted decellularized meniscus after 1 (**A**) and 4 weeks (**B**). The histological observation confirmed the biocompatibility of the scaffold. Arrows show meniscus residual

Inflammatory and immune responses of the decellularized scaffold were evaluated 1 and 4 weeks after subcutaneous implantation. On week 1, a large number of monocytes and neutrophils were observed around the implantation area, but the number of the immune cells decreased extensively 4 weeks after implantation. After 1 week, there was no evidence of angiogenesis around the implanted scaffold. The scaffold was partially degraded 4 weeks after implantation and surrounded by vascular area (Fig. 4A–B).

### ECM preservation

**Fig. 5:**
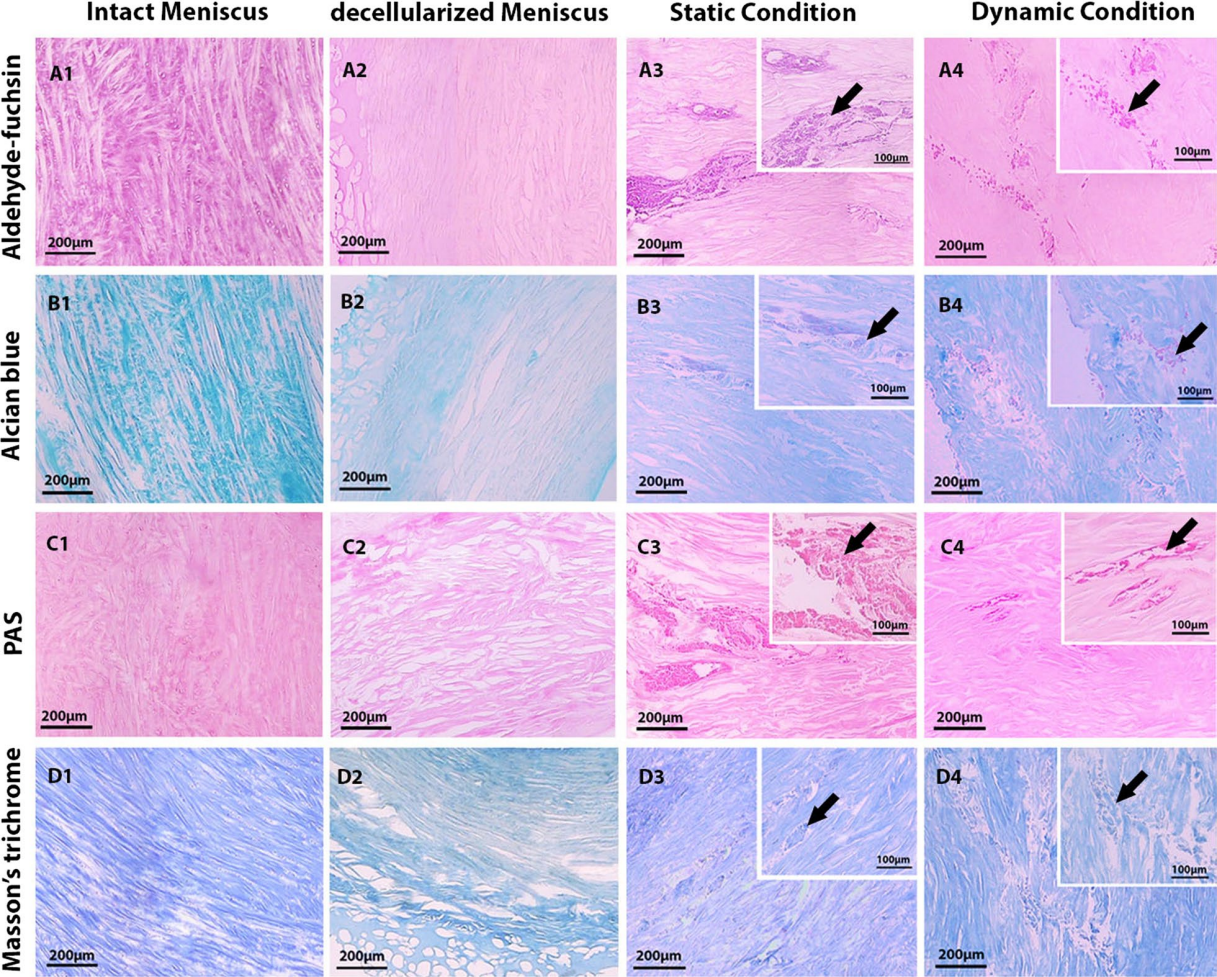
Aldehyde-fuchsin (**A**1–**A4**), Alcian blue (**B**1–**B**4), periodic acid Shift (**C**1–**C**4), Massons’ trichrome (**D**1–**D**4) staining of the native, decellularized, and recellularized menisci under static and dynamic conditions. Arrows indicate the chondrocytes seeded on decellularized menisci under static and dynamic condition

Gomori's aldehyde-fuchsin, Alcian blue, and PAS staining revealed that the GAG content, elastic fibers, and neutral carbohydrates were slightly decreased in the decellularized scaffold and increased again after recellularization and mechanical stimulation (Fig. 5 A1–A4, B1–B4, C1–C4).

Masson’s trichrome staining revealed collagen fiber retention in both decellularized and recellularized scaffolds with and without mechanical stimulation (Fig. 5 D1–D4).

### Degradation and hydration rate

**Fig. 6:**
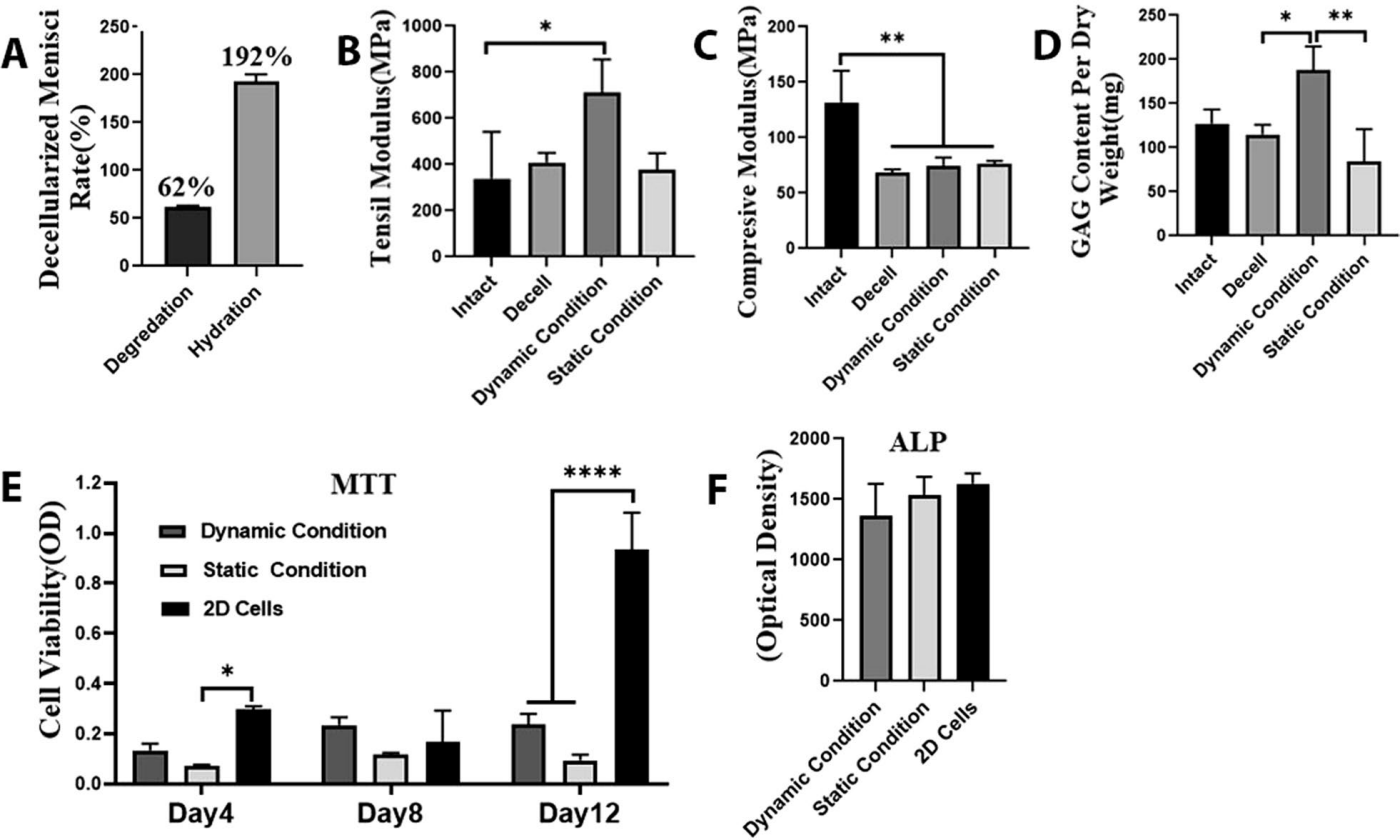
**A**, degradation and hydration rate of decellularized scaffold; **B**, Tensile (*p < 0.05) and (**C**) compression (**p < 0.01) moduli of the native, decellularized and recellularized menisci under static and dynamic conditions. **D**, GAG content per dry weight (100 mg, *p < 0.05, **p < 0.01). **E**, MTT test showed viable cells are present in all the conditions (*p < 0.05, ****p < 0.001). **F**, Alkaline phosphatase activity in 2D and 3D conditions with or without mechanical stimulation

The in vitro degradation of lyophilized decellularized scaffolds was expressed as a 62% mass loss after 21 days. The hydration rate of the scaffolds was a 192% increase in the weight in a 4 h immersion in distilled water (Fig. 6A).

### Mechanical testing

Mechanical tests were used to mechanically quantify robust recellularized meniscal scaffold when subjected to mechanical loading. Tensile modulus in the mechanical stimulated group was significantly higher (710 ± 83.7 MPa) compared to intact ones (339 ± 116.6 MPa, *P* = 0.03). The scaffolds in dynamic condition had a non-significant higher tensile strength than the decellularized menisci (409.2 ± 23.9 MPa) and static condition (375.2 ± 42.8 MPa, Fig. 6B). The result demonstrated that collagen fibers and other components of ECM worked well to provide appropriate tensile properties in the recellularized menisci under mechanical loading; however, the compressive modulus was significantly decreased in decellularized menisci and cell-laden scaffolds under both dynamic and static conditions compared to the intact menisci (*P* = 0.003, *P* = 0.006, and *P* = 0.008, respectively, Fig. 6C).

### GAG quantification test

GAG content significantly increased in the mechanical stimulated scaffolds compared to the decellularized and non-stimulated scaffolds (*P* = 0.02, *P* = 0.003, respectively), so the amount of GAG deposition in dynamic condition was as high as that in the intact tissue (*P* = 0.2, Fig. 6D).

### Cytotoxicity assay

Chondrocytes were seeded into the decellularized meniscal scaffold under static and dynamic stimulation and the cell viability and proliferation were assessed using the MTT test and compared to 2D monolayer condition after 4, 8, and 12 days. On day 4, in the 2D group, the proliferation rate was remarkably higher than the static condition (*P* = 0.01). On day 8, viability and proliferation were the same in both 3D and 2D conditions. On day 12, in 2D condition, proliferation was significantly higher than both 3D conditions (*P* = 0.001, Fig. 6E).

### Alkaline phosphatase activity

The ALP activity in the cell-laden scaffold was non-significantly lower than the 2D condition, and there was no evidence of mineralization in the recellularized meniscus under both static and dynamic conditions (Fig. 6F).

### Real-time RT-PCR

**Fig. 7:**
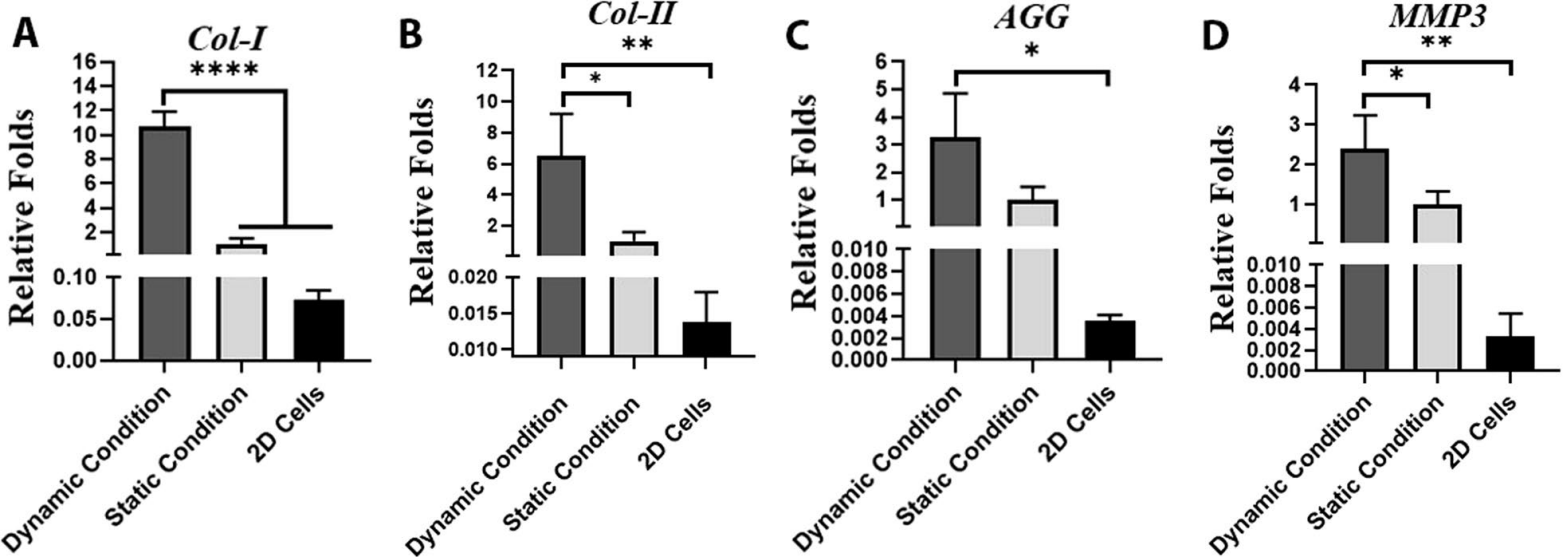
Expression of **A** COL-I, **B** COL-II, **C** AGR, and **D** MMP3 by chondrocytes cultured in decellularized meniscus under static and dynamic conditions compared to 2D conventional condition (*p < 0.05, **p < 0.01 and ****p < 0.001)

The expression of *COL-I*, *II*, *AGR*, and *MMP3* in recellularized meniscal scaffold under static and dynamic stimulation was quantified using RT-PCR and compared to the 2D conventional conditions with the same number of chondrocytes as the control group. The results revealed a significantly higher expression level of *COL-I* (*P* = 0.00005), *COL-II* (*P* = 0.006), *AGR* (*P* = 0.01), and *MMP3* (*P* = 0.003) in meniscal scaffolds under dynamic conditions, compared to the 2D conventional conditions. However, the expression level of these genes was statistically the same in the cells cultured in static 3D decellularized and 2D conditions, indicating that decellularized scaffolds its own had a minor role in boosting the cartilage-specific marker expression and subjecting to mechanical load can improve tissue specific marker expression. The expression of *COL-I* (*P* = 0.00009), *II* (*P* = 0.06), *AGR* (*P* = 0.01), and *MMP3* (*P* = 0.03) under dynamic compression and shear loading increased 10.7-folds, 6.4-folds, 3.2-folds, and 2.3-folds compared to the static condition, respectively. These observations suggest that mechanical stimuli mimic the joint mobilization and cause the chondrocytes to create their surrounding matrix (Fig. 7).

### Immunohistochemistry

**Fig. 8:**
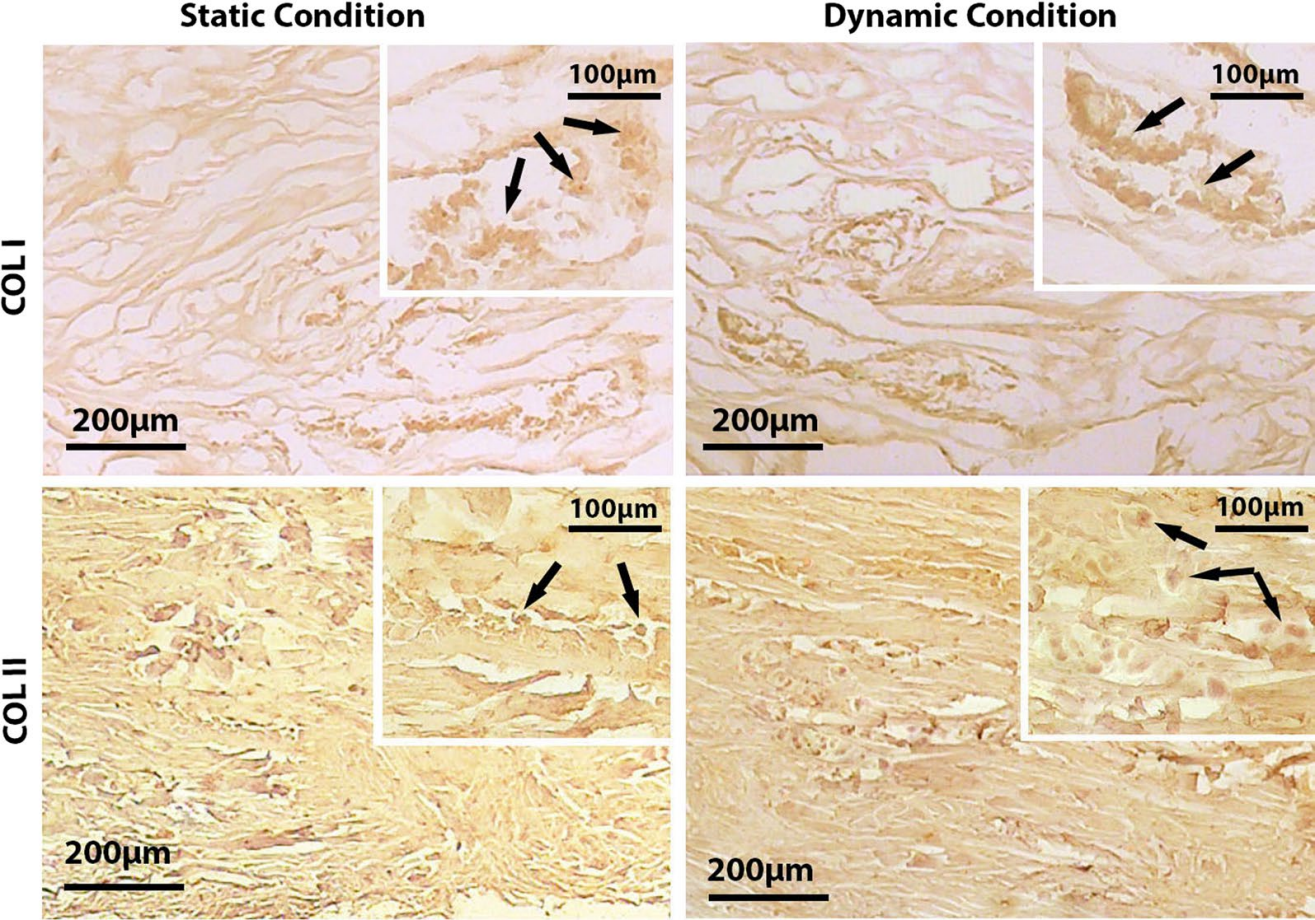
Immunohistochemical staining of COL-I and II in decellularized meniscus under static and dynamic conditions. Arrows indicate the chondrocytes seeded on decellularized meniscus

Immunostaining confirmed an increase in the COL-I after mechanical stimulation compared to the static condition (Fig. 8).

### Raman spectrum

**Fig. 9:**
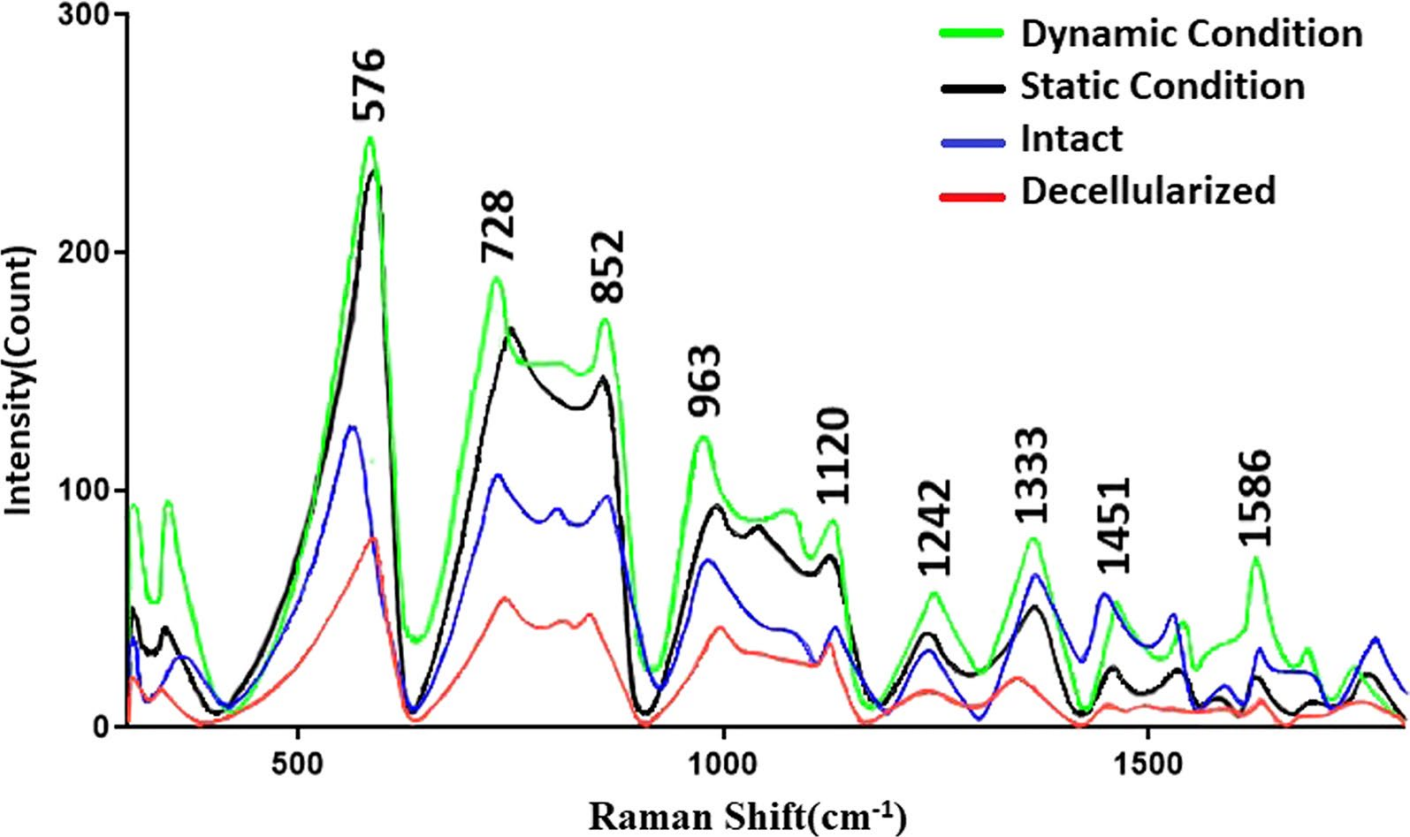
Raman spectra of recellularized scaffold under dynamic and static conditions were compared with intact and decellularized menisci. Existence of the main collagen bands, amide III, RNA and DNA content was detected

Raman confocal spectrometry revealed the trend of the Raman spectra was the same in all the scaffolds and they have similar trend as intact meniscus. The intensity of the Raman spectra of the decellularized scaffolds was the least compared to all the groups that may indicate the washing of the content or producing ECM content after cell loading. A bond at 963 cm^−^1 indicated unassigned protein. The peaks in 728 cm^−1^, 852 cm^−1^, 1451 cm^−1^ and 1586 cm^−1^ were assigned for proline and hydroxyprolin that are specific amino acids in collagen. A peak at 1242 cm^−1^ assigned for amide III. Vibration at 1120 cm^−^1 and 1333 cm^−1^ represented strong C-O bond of ribose in RNA and guanine, respectively. A peak at 576 cm^−1^ indicated phosphatidylinositol that present in cell membrane (Fig. 9) [25].

## Discussion

To the best of our knowledge, this is the first study which used a cell-laden whole decellularized meniscus subjected to compress and shear stimulation for recapitulating the meniscus. A suitable biocompatible meniscus ECM preserves the biomechanical properties of the native tissue [26]. Subcutaneous implantation of a porous decellularized porcine meniscus has been reported to be biocompatible without any sign of immune response. Human chondrocyte-seeded scaffold showed an enhancement in cell proliferation and ECM synthesis including COL-II and GAG [27]. Collagen fibers and GAG retention as well as appropriate biomechanical properties have been reported in decellularized whole porcine [28] and ovine [10] meniscus. Decellularized porcine meniscus ECM provided an appropriate non-toxic surface for the fibroblasts and chondrocytes attachment and infiltration [29]. Besides, increase in porosity after decellularization supported stem cell viability and proliferation without cellular toxicity [28]. Biocompatibility without considerable immune response was confirmed by the current study after 4 weeks of subcutaneous implantation of the decellularized meniscus of rabbit. An in vitro study also indicated that the scaffold was non-toxic. On the other hand, a previous study showed that the porosity was increased by peracetic acid and triton-X100 administrations, and it facilitated the diffusion of nutrients to the interior pores and promoted the cell growth and proliferation. The porosity has a positive correlation with hydration [27]. We also confirmed that these reagents elevated the scaffold hydration (192%) and cell proliferation followed by an increase in porosity. The data from the current study indicated an increase in the chondrocyte function by culturing in the decellularized meniscus. Recellularization of the decellularized porcine menisci with human chondrocytes has been detected to promote the cell proliferation and provide chondrocyte ECM including COL-II and GAG [27]. Cell lines and primary chondrocytes have been used widely to assess the cellular compatibility of the scaffolds and their potential operation for regeneration and repair of the cartilage tissue and meniscus [30, 31]. Decellularized ovine meniscus was recellularized by manual injection of the autologous fibrochondrocytes. This technique allowed the cells to penetrate completely into the center of the scaffold without any evidence of toxicity over 4 weeks [32]. Our data also confirmed that cell injection could be considered an appropriate procedure for recellularization. Weight-bearing is an important factor in the arrangement of the collagen fibers and chondrocytes, as well as the shape, size, and biochemical properties of the cartilage ECM [33, 34]. Many different bioreactors have been designed to apply compression and shear forces or combinations of them in a controlled environment [14] and mimic the effects of the mechanical loading during development. Our ad hoc bioreactor also induced a combination of the compress and shearing forces without a significant impression on the cell viability. Some research was conducted to find the effects of mechanical stimulation on the cells encapsulated in the various scaffolds. Compression and shear tension on the engineered articular cartilage [35] and, meniscus [36] have been also shown to preserve the cellular viability and proliferation. Cyclic compression on the chondrocyte-laden hydrogel fabricated by collagen-I promoted the cell functions and viability [19]. Besides, viable chondrocytes and integrated scaffolds were detected after exposing the agarose constructs to cyclic compression and shear at 1 Hz for 48 h [37]. Our data also confirmed that mechanical stimulation on the chondrocyte-loaded decellularized scaffolds had no detrimental impact on the cell viability. The bovine decellularized meniscus also provides a good platform for fibrochondrocytes to spread, proliferate, and produce GAG and collagen [38]. A higher level of aggrecan and COL-II was reported by culturing the synovial MSCs on different regions of the porcine decellularized meniscus. The cells in different regions expressed zone-specific markers as well. Besides, subcutaneous implantation of cell-laden decellularized meniscus showed a higher level of GAG production [11]. In line with these studies, we also found a significant elevation in the GAG production and COL-I, COL-II, aggrecan, and MMP3 expression by culturing the cells in the decellularized meniscus. Meniscus fibrochondrocytes on a collagen scaffold were stimulated by 30 and 40% strain over 6 weeks. The findings revealed upregulation of the expression of hyaline cartilage markers such as SOX9 and COL-I [39]. Dynamic compression on the chondrocytes encapsulated in a viscoelastic hydrazone scaffold showed a higher level of matrix biosynthesis, including the GAGs and collagen [40]. In another study, 10% strain of dynamic mechanical compression at 1 Hz for 1 h/day for 2 weeks on chondrocyte-laden silk fibroin scaffolds induced GAG and matrix accumulation and expression of aggrecan and COL-X [41]. Mechanical loading also improved the fibrochondrocyte differentiation of Bone Marrow Mesenchymal Stem cells (BMSCs) seeded in fibrin constructs, as indicated by increases in the expression of *COL-I* [42]. These findings are in agreement with our data that indicates a higher level of aggrecan, COL-I, and II by exposing the cells to mechanical stimulation. Raman confocal spectrophotometry also confirmed that collagens increased in dynamic state compared to decellularized meniscus. Several studies reported the effects of dynamic shear loading (0.01–1.0 Hz, using 1–3% strain) on chondrocyte biosynthesis and showed an increase in cartilage matrix components including GAG, COL-I and II, aggrecan, and matrix metalloproteinase [43]. In another study, a combination of mechanical stimulation (shear and compression) was applied to the cell-laden engineered de novo cartilage-like tissue. A loading force of 0.5 N and 0.276 Hz caused an increase in COL-II and aggrecan mRNA and enhanced the GAG/DNA content [44]. Our data also confirmed the previous studies.

We found that delayed mechanical stimulation led to an increase in MMP3 expression. MMP3 has a role in matrix degradation and remodeling [45]. The scaffold constitution and time of applying mechanical stimulation were found to be important in regulation of MMP3 expression. For instance, immediate application of intermittent compression of human chondrocytes in poly- (ethylene glycol) hydrogel led to an increase in MMP3 expression, while 1 week delay on force administration led to a decrease in MMP3 expression [46]. In contrast to this finding, MMP3 was not changed by application of the dedifferentiated osteoarthritic human chondrocytes encapsulated in COL-I hydrogel [47]. These contradictory results may indicate that cell response to mechanical stimulation depends on the culture condition including the type of scaffold and the time of force administration. Besides, we applied a simultaneous stimulation of shearing and compression forces.

Static mechanical load inhibits the matrix synthesis and cell proliferation, while dynamic loading promotes the ECM synthesis. Besides, low dynamic compression (1–5% and 0.01 Hz) increased the metalloproteinase activity (MMP-2, MMP-9) [48]. Under the dynamic condition, the chondrocytes are differentiated into the fibrochondrocytes that synthesizes COL-I rather than COL-II. This dedifferentiation process can be related to the alternation in the cytoskeletal framework, shape, and size of the chondrocytes through mechanotransduction processes. This condition also played a main role in the organization of the collagen fibers within the ECM (naturally or in an explanted scaffold). Although in the current study, static stimulation was not applied, a combination of compression and shearing led to an increase in the production of the matrix. The mechanical stimulation also elevated the tensile strength. This may be attributed to an increase in COL-I biosynthesis [49]. Our findings revealed that dynamic stimulation enhanced the tensile strength along with COL-I.

Orientation of the collagen fibers was preserved after decellularization of porcine meniscus ECM [12, 29]. Normal load-bearing on the meniscus is an important factor in determining the orientation of collagen fibers [33], and the fiber orientation was enhanced under dynamic mechanical stimulation rather than static condition [50]. Our data indicated that the arrangement of collagen fibers was maintained in the whole decellularized meniscus, but with slight disorientation. We also showed that the fibers were more aligned in the recellularized scaffold under dynamic stimulation compared to static condition. The decellularization led to the formation of micropores in the collagen bundles that made the fibers more incompact and thickened [32, 51] than the native meniscus. Furthermore, the accumulation and density of collagen fibers increased after cell loading and mechanical stimulation [16, 50]. In agreement with these reports, the thickness of the collagen fibers in our decellularized meniscus was significantly higher than that in intact, dynamic, and static conditions. It seems that cell seeding and mechanical stress enhanced the compaction and reduced the collagen fiber thickness.

An in vivo study revealed that abnormal mechanical stimulation on the bovine meniscus enhanced the mineralization markers [52]. Our data showed that the dynamic mechanical loading on recellularized scaffolds had no sign of calcification, which may be due to the similarity of the mechanical stimulation with normal loading in the naïve meniscus.

In this study, we designed a bioreactor that simultaneously applied both compression and shearing forces to the scaffolds. Mechanical forces have a critical role in the cell metabolism [53]. Besides, periodic mechanical forces mimic the walking in human. In the current study, 1 HZ compression was applied in human chondrocytes loaded in the rabbit scaffold that is comparable to waking in human [54]. This ad hoc designed bioreactor also mimics rotational movement of the joint such as knee joint. In human, the rotational movement in joints is about 25° [21] and the bioreactor also produces a 25° rotational shearing movement.

The most important limitation of this study was to use a cell line instead of primary cells. Although the regulations for commercialization and shipping of the allogenic primary cells are more restricted, the transplantation of the engineered meniscus contained cell line is impractical.

## Conclusion

We demonstrated that the decellularized ECM scaffold provided a suitable microenvironment for chondrocyte activities. Furthermore, ad hoc designed bioreactor was able to apply simultaneous compress and shear forces and mimic a normal load-bearing meniscus during the joint movement. Encapsulating the chondrocyte on the decellularized scaffold responded to the dynamic loading by increasing the tensile strength and ECM production.

## Data Availability

All data are available in the current article.

## Abbreviations

MTT: 3-(4, 5-Dimethyl thiazolyl-2)-2, 5-diphenyltetrazolium bromide
ANOVA: Analysis of variance
AGG: Aggrecan
COL-I: Collagen-I
DAB: Diaminobenzidine
DMMB: Dimethyl methylene blue
DMEM: Dulbecco's modified eagle medium
EDTA: Ethylenediaminetetraacetic acid
ECM: Extracellular matrix
FBS: Fetal bovine serum
GAG: Glycosaminoglycan
H&E: Hematoxylin and eosin
MMP3: Matrix metalloproteinase
MSCs: Mesenchymal stem cells
PAS: Periodic acid schiff
PBS: Phosphate buffer saline
SEM: Scanning electron microscopy
SLES: Sodium lauryl ether sulfate
